# Secretarybird *Sagittarius serpentarius* Population Trends and Ecology: Insights from South African Citizen Science Data

**DOI:** 10.1371/journal.pone.0096772

**Published:** 2014-05-09

**Authors:** Sally D. Hofmeyr, Craig T. Symes, Leslie G. Underhill

**Affiliations:** 1 School of Animal, Plant and Environmental Sciences, University of the Witwatersrand, Johannesburg, Gauteng, South Africa; 2 Animal Demography Unit, Department of Biological Sciences, University of Cape Town, Cape Town, Western Cape, South Africa; University of KwaZulu-Natal, South Africa

## Abstract

Data from two long-term citizen science projects were used to examine the status and ecology of a Red List species, the Secretarybird *Sagittarius serpentarius* (Vulnerable), in South Africa. The first phase of the Southern African Bird Atlas Project operated from 1987 until 1992, and the second phase began in 2007. The Coordinated Avifaunal Roadcounts (CAR) project began in 1993 and by 1998 had expanded to cover much of the south-eastern half of the country. Data submitted up until April 2013 were used. A new method of comparing reporting rates between atlas projects was developed. Changing reporting rates are likely to reflect changes in abundance; in this instance the data suggest that the Secretarybird population decreased across much of South Africa between the two atlas projects, with a widespread important decrease in the Kruger National Park. Habitat data from the CAR project were analysed to gain insight into the ecology of the species. Secretarybirds tended to avoid transformed habitats across much of the area covered by the CAR project. In the winter rainfall region of the Western Cape, which is characterised by heavily transformed fynbos vegetation, at least 50% of Secretarybirds recorded were in transformed environments. This implies that in the Fynbos biome, at least, Secretarybirds have adapted to transformed environments to some degree. However, in the rest of the country it is likely that habitat loss, largely through widespread bush encroachment but also through agriculture, afforestation, and urbanisation, is a major threat to the species. The methods developed here represent a new approach to analysing data from long-term citizen science projects, which can provide important insights into a species' conservation status and ecology.

## Introduction

The global population of Secretarybirds *Sagittarius serpentarius* is in decline, and the species was classified as Vulnerable internationally on the IUCN Red List in 2011 [1]. This species is generally thinly distributed under normal conditions, and exhibits varying degrees of nomadism, depending on local conditions [2]. The probability of seeing these birds is generally relatively low or at least variable, which makes the species difficult to census reliably using standard count methodologies [3], and gradual changes in abundance may remain undetected for some time.

Secretarybirds occur throughout much of sub-Saharan Africa, with the exception of forested west Africa and the Horn of Africa [2], [4]. Their preferred habitat is grassland, dwarf shrubland, savanna, and open woodland; they avoid forest, thicket, dense woodland and rocky, mountainous or hilly areas [2], [5]. Secretarybirds are monogamous and territorial; they nest on the tops of small, dense trees and usually raise one or two chicks, occasionally three, per breeding attempt [2]. Breeding in South Africa occurs throughout the year, with a peak from the austral late winter to early summer [2]. In arid areas birds are nomadic when not breeding; the more mesic the habitat, the more sedentary the birds tend to be, but when they are not breeding they usually do at least display increased local movements [2], [5].

Long-term public participation (“citizen science”) projects make it possible for observations made by many different people to be pooled and analysed as a whole [6]–[10]. They provide the best opportunity for assessing population trends in species such as the Secretarybird. The first and second Southern African Bird Atlas Projects (SABAP1, 1987–1992, and SABAP2, 2007–present) offer two snapshots of avian distribution in South Africa approximately 15 years apart [11], [12]. The Coordinated Avifaunal Roadcounts (CAR) project (described below), was established in the Western Cape in 1993, by 1998 had expanded to cover much of South Africa [13], and in 2014 was ongoing. We examine the information provided by these datasets for useful insight into the status and ecology of Secretarybirds.

We develop a method for inferring changes in abundance from atlas reporting rates, with a measure of statistical significance attached. While the count data provided by the CAR project are not useful for a species such as the Secretarybird, because of its low general abundance and nomadic/wide-ranging behaviour [3], the habitat use data provide information about the species' ecology. Together, these analyses provide important insights into the conservation status of the Secretarybird in South Africa in 2013, which would not have been possible without the existence of these citizen science data.

## Methods

### SABAP

#### Data collection

The first and second Southern African Bird Atlas Projects (SABAP1 and SABAP2) represent distinct iterations of southern Africa's largest-scale bird monitoring project [12]. The protocol used for SABAP1 (1987–1992) was described in detail by Harrison and Underhill [14], and for SABAP2 (2007–present) on the project's website (http://sabap2.adu.org.za/). For each atlas project, checklists were collected for grid cells throughout South Africa. SABAP1 used quarter degree grid cells (QDGCs, 15'×15') and SABAP2 used 5'×5' grid cells (pentads). Checklists could be collected over periods of up to one month for SABAP1 and up to five days for SABAP2, but in practice most checklists for both projects were collected on a single day.

#### SABAP comparison map

We used a visual method, termed a SABAP comparison map, to display apparent changes in abundance between SABAP1 and SABAP2. Our starting point was the reporting rates, the proportion of checklists which reported the species in a particular grid cell, in each of the atlas projects. For SABAP2, we used data submitted to the project up until 19 April 2013. Although there are caveats to the interpretation of reporting rates [14], there is strong evidence that reporting rates are monotonically related to abundance, albeit in a non-linear manner (see Discussion, and e.g. [12], [15]–[20]). For a single species in one atlas grid cell, the change in reporting rates between projects is most likely to be attributable to changes in abundance, especially when the change in reporting rate is substantial, and the numbers of checklists on which the reporting rates are based is large. We used the standard statistic for the test for equality of two proportions [21] as the basis for assessing whether the observed data were likely to represent real change. We chose this formula because it contains the three key quantities (change in reporting rates and the two sample sizes) used in a theoretically understood environment. When used in the statistical hypothesis testing framework, this statistic has, asymptotically, the standard normal distribution. This provides a first approach to interpreting whether a change in reporting rates is statistically significant, taking into account the numbers of checklists which are available. The statistic was calculated as follows 
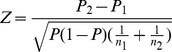
 where *P*_1_ and *P*_2_ are the reporting rates from SABAP1 and SABAP2 respectively, *n*_1_ and *n*_2_ are the numbers of checklists on which the reporting rates are based, and *P* is the pooled reporting rate



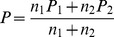
.

**Figure 1:**
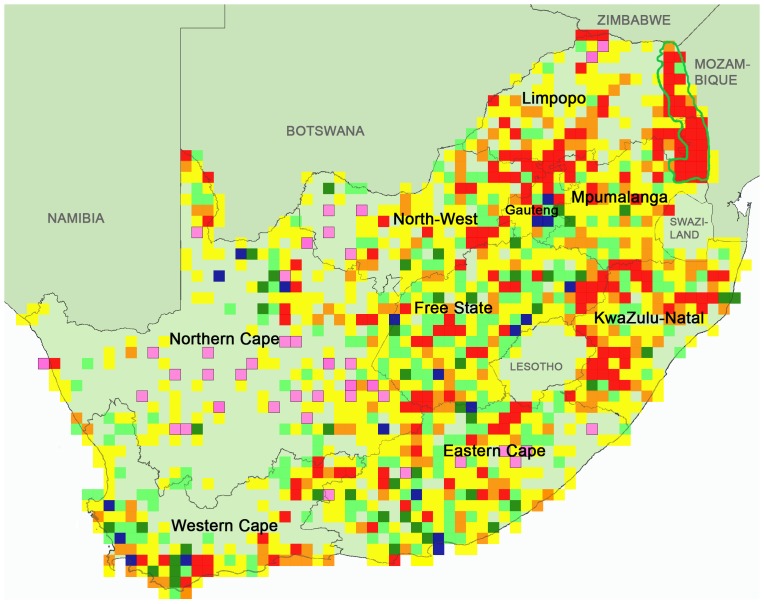
Southern African Bird Atlas Project (SABAP) comparison map for the Secretarybird, extracted 19 April 2013. This map compares SABAP1 and SABAP2 reporting rates. South African province names are given in black, neighbouring countries are labelled in grey, and the Kruger National Park, in the north-east of South Africa, is outlined in green. Coloured squares are quarter-degree grid cells (QDGCs; 15'×15') in which the species was observed in either project. Reporting rates are compared using the *Z*-statistic (see text). SABAP2 reporting rates were lower than SABAP1 in red, orange and yellow grid cells, and higher than SABAP1 in light and dark green and blue grid cells. In red grid cells *Z*<–2.58 (important decrease), in orange –2.58<*Z*<–1.64 (distinct decrease), and in yellow –1.64<*Z*<0 (decrease probably attributable to sampling variability). In light green grid cells 0≤*Z*<1.64 (increase probably attributable to sampling variability), in dark green 1.64<*Z*<2.58 (distinct increase), and in blue grid cells *Z*>2.58 (important increase). Pink grid cells are those which had not yet been covered in SABAP2. Therefore, red, orange and yellow grid cells indicate areas of potential conservation concern, whereas green and blue grid cells indicate areas of apparent population increase.

These results are presented as a map, which facilitates spatial interpretation and enables us to highlight areas of concern. We used a six-colour system to classify the *Z*-score for each QDGC into categories, using familiar values from the standard normal distribution as the cutpoints for the *Z*-scores, but without associating the usual significance levels with them (Figure 1). An additional category was created for QDGCs in which the species was recorded in SABAP1 but for which no checklists had been submitted for SABAP2 at the time of the data download.

Although we are making use of theory and values associated with hypothesis testing, these comparison maps should be seen as a tool of exploratory data analysis (in the sense of Tukey [22]), i.e. a tool that enables us to balance the importance of a difference in proportions (reporting rates) in a grid cell against the sample sizes (numbers of checklists) that generated them.

### CAR project

#### Data collection

**Figure 2:**
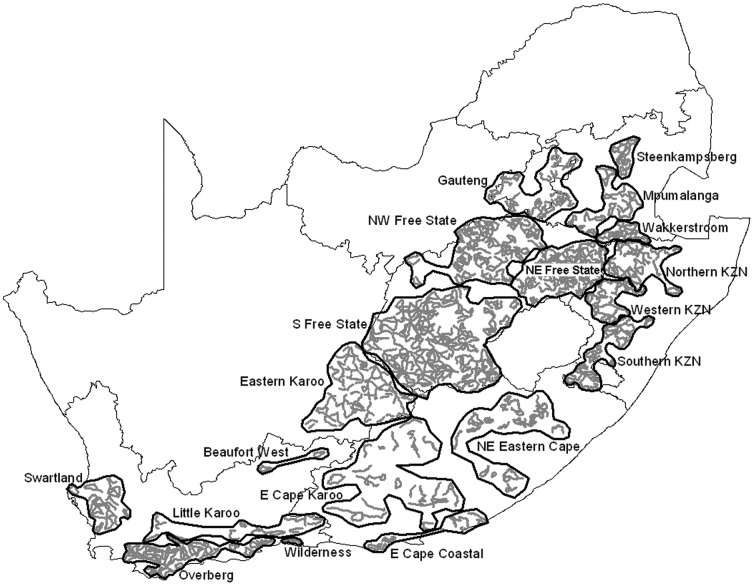
Coordinated Avifaunal Roadcounts (CAR) project survey routes and precincts. Routes are indicated by thick grey lines, and precincts are outlined in black. CAR routes covered c. 19 000“KZN”, KwaZulu-Natal. Precincts were defined on the basis of ecological characteristics by Young et al. [13] (within precincts the natural vegetation type and climatic conditions are more similar than between precincts) and precinct names follow Young et al. [13].

The fieldwork for the CAR project consisted of six-monthly (biannual) counts of large terrestrial birds along roads through agricultural areas across approximately half of South Africa [13]. Participants drove slowly (not faster than 50 km h^–1^) along fixed routes and stopped every 2 km to get out of the vehicle and scan the area with binoculars, counting every large terrestrial bird they saw. They also stopped, scanned for, and counted birds if they saw any of the target species between the 2 km stops. The project was initiated in 1993 in the Overberg region of the Western Cape and expanded to cover much of the south-eastern half of South Africa over the following eight years (Figure 2) [3], [13]. Secretarybirds were included in the list of species surveyed from 1995 onwards. Data from surveys up until summer 2013 have been included in these analyses.

The routes, which were c. 60 km long, were surveyed twice a year, in summer (last Saturday in January) and in winter (last Saturday in July). Note that seasons are austral. When counting birds, participants also collected other relevant variables, including the habitat in which the bird was seen, age class of birds, and weather conditions on the day of the count. The habitat in which the bird was seen was ascribed to one of several categories; those relevant to this analysis are “veld” (natural vegetation), pasture, crop fields, fallow land, crop stubble (harvested fields with only the crop residue remaining), water (any type of water body including man-made water points) and a catch-all category called “agric land”, which included anything that was part of farmland (in this case mainly ploughed, burned, and mowed land) but didn't fall into any of the other prescribed categories. These categories were chosen to cater for the dual requirements that they be (a) identifiable by laypeople, and (b) relevant to large terrestrial birds. Most of the project participants were members of bird clubs, were farmers or residents of the area in which they conducted their surveys, and surveyed the same route for the duration of their participation in the project. They were thus familiar with the area and could identify bird species, especially Secretarybirds, and habitats, reliably.

#### Habitat use

Habitat use data collected by CAR project participants were extracted and summarised. Routes were classified into “precincts” — ecologically distinct areas with similar vegetation and climate characteristics — following Young et al. [13] and based on broad scale vegetation types [23] of areas surrounding any new routes. Precincts were included if the number of routes on which birds were observed in at least one of the seasons was five or more, irrespective of the number of birds observed.

CAR project participants collected data at the locations at which Secretarybirds were present and not in the landscape as a whole. The National Land-Cover maps for 1994, 2000 and 2009 provided consistent data covering the entire study area for the time period in question (NLC1994, NLC2000 and NLC2009 respectively) [24]–[26].

Habitat selection in relation to habitat availability was analysed by comparing the proportions of natural and transformed land available to the proportions of birds seen in each type of land in each precinct. The proportions of natural and transformed land available were calculated using all three NLC maps. All classes of land other than natural vegetation classes and waterbodies were combined to form a single “transformed” land class, and the remaining categories were combined to form a “natural” class (man-made and natural waterbodies, including wetlands, are not distinguished in the maps). A caveat to this analysis is the distinction between transformed, modified, and natural land [3]. Land classified as “transformed” is that on which the vegetation structure has been completely altered, e.g. pastures and cultivated fields. The “natural” habitats mapped in the National Land-Cover maps are in fact mostly modified habitats, i.e. habitats in which the overall structure and main components of the natural vegetation remain, but in which important changes have been made, e.g. the naturally occurring community of herbivores has been replaced by livestock. In 1989 it was estimated that only 7% of South Africa's land surface remained fully natural and undisturbed [27]. Thus in these analyses when we refer to natural habitats, we are in fact mostly referring to modified habitats. However, the extent to which they had been modified and to which they differed functionally from natural habitats, from the perspective of a Secretarybird, would be variable.

Allan [28] considered that observers using binoculars can detect Blue Cranes *Anthropoides paradiseus* up to a maximum distance of 1 500 m away. Because Secretarybirds are a similar size, shape and colour, we assume that the same applies to them. To calculate the proportion of natural to transformed habitats available to Secretarybirds and visible from the road, we used ArcView 3.1 [29] to form a buffer zone of width 3 000 m along each CAR route (i.e. 1 500 m on each side of the road), which was overlaid with the transformed/natural layer produced from the NLC maps. The percentages of transformed and natural land were calculated for each route for each NLC map, and this was compared with the percentages of birds seen in each habitat type on each route, using the Jacobs index [30]. Jacobs index values range between +1, indicating total positive selection, and –1, indicating total negative selection. The “sign test” was used to evaluate whether the number of routes in a precinct for which the index value was positive differed significantly from the number for which it was negative [31].

These habitat selection analyses were also performed by province (as opposed to precinct), to facilitate easier comparison with the SABAP data, which was summarised by province. Habitat use data from the CAR project were also summarised graphically by precinct to gain some insight into the types of transformed land used by Secretarybirds in different areas.

## Results

### SABAP

**Figure 3:**
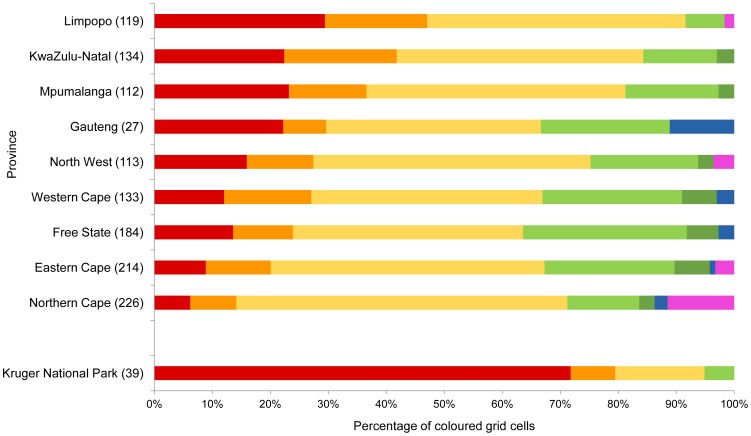
Numbers of quarter degree grid-cells (QDGCs) of different categories per province in Figure 1. Numbers in brackets following the names of the provinces are the numbers of QDGCs in which Secretarybirds were ever recorded. SABAP2 reporting rates were smaller than SABAP1 in red, orange and yellow grid cells, and greater than SABAP1 in light and dark green and blue grid cells (see Figure 1). Pink grid cells are those which had not yet been covered in SABAP2.

As at 19 April 2013 Secretarybirds had been reported in 1 262 QDGCs across South Africa, 64.9% of all South African QDGCs (Table 1, Figures 1 and 3). The Northern Cape had 26 QDGCs (11.5% of the provincial total of 226) for which Secretarybirds had been reported in SABAP1 but which had not yet been visited for SABAP2. For the country as a whole this figure was 39 (3.1%), with Limpopo, North-West and Eastern Cape contributing two, four and seven such QDGCs respectively.

**Table 1:** Counts of quarter degree grid cells (QDGCs, 15'×15') in the Southern African Bird Atlas Project (SABAP) comparison map for Secretarybirds for 19 April 2013 (Figure 1).

Province	Red QDGCs	Orange QDGCs	Yellow QDGCs	Light Green QDGCs	Dark Green QDGCs	Blue QDGCs	Pink QDGCs	Total QDGCs	Non-Zero SABAP1 QDGCs	Zero SABAP2 QDGCs	Proportion of non-zero SABAP1 QDGCs that were zero in SABAP2 (%)	Proportion of total QDGCs that were zero in SABAP1 (%)
Northern Cape	6.2 (14)	8.0 (18)	57.1 (129)	12.4 (28)	2.7 (6)	2.2 (5)	11.5 (26)	226	207	125	60.4	8.4
Eastern Cape	8.9 (19)	11.2 (24)	47.2 (101)	22.4 (48)	6.1 (13)	0.9 (2)	3.3 (7)	214	194	91	46.9	9.4
Free State	13.6 (25)	10.3 (19)	39.7 (73)	28.3 (52)	5.4 (10)	2.7 (5)	0.0 (0)	184	166	48	28.9	9.8
Western Cape	12.0 (16)	15.0 (20)	39.8 (53)	24.1 (32)	6.0 (8)	3.0 (4)	0.0 (0)	133	111	50	45.1	16.5
North West	15.9 (18)	11.5 (13)	47.8 (54)	18.6 (21)	2.7 (3)	0.0 (0)	3.5 (4)	113	102	54	52.9	9.7
Gauteng	22.2 (6)	7.4 (2)	37.0 (10)	22.2 (6)	0.0 (0)	11.1 (3)	0.0 (0)	27	24	2	8.3	11.1
Mpumalanga	23.2 (26)	13.4 (15)	44.6 (50)	16.1 (18)	2.7 (3)	0.0 (0)	0.0 (0)	112	109	50	45.9	2.7
KwaZulu-Natal	22.4 (30)	19.4 (26)	42.5 (57)	12.7 (17)	3.0 (4)	0.0 (0)	0.0 (0)	134	125	38	30.4	6.7
Limpopo	29.4 (35)	17.6 (21)	44.5 (53)	6.7 (8)	0.0 (0)	0.0 (0)	1.7 (2)	119	117	74	63.3	1.7
Totals	15.0 (189)	12.5 (158)	46.0 (580)	18.2 (230)	3.7 (47)	1.5 (19)	3.1 (39)	1262	1155	532	46.1	8.5

QDGCs are coloured if the species was observed there in either project. Reporting rates are compared using a *z*-statistic (see text). SABAP2 reporting rates were lower than SABAP1 in red, orange and yellow grid cells, and higher than SABAP1 for light and dark green and blue grid cells (see Figure 1). Pink grid cells are those that had not yet been covered in SABAP2. Therefore, red, orange and yellow grid cells indicate areas of potential conservation concern, whereas green and blue grid cells indicate areas of apparent population increase. Columns labelled with colours give the percentage of the total for that province, with the actual number of QDGCs in parentheses. Columns to the right of the Total column present summaries of reporting rate data not presented in Figure 1 but available in Figure S1.

The SABAP2 reporting rate had decreased relative to SABAP1 reporting rates in 927 (73.5%) of these QDGCs, and this decrease was important (red and orange QDGCs, Z<–1.64) in 347 QDGCs (27.5%; Table 1, Figures 1 and 3). In every province, reporting rates had decreased in more than 60% of the QDGCs in which the species had been reported in either project (Figure 3). The province with the greatest proportion of QDGCs in which reporting rates decreased was Limpopo (91.6%). Free State had the smallest proportion, 63.6%. Limpopo also had the largest proportion of important decreases: 47.1%, while Northern Cape had the smallest: 14.2%.

Of the 39 QDGCs including any area inside the Kruger National Park, South Africa's largest conservation area, reporting rates decreased in 37 (94.9%). This decrease was important in 31 QDGCs (79.5%). As at 19 April 2013 these 39 QDGCs had an average of 131.9 lists per QDGC for SABAP1 and 111.8 for SABAP2, compared with an average of 42.9 lists per QDGC for SABAP2 for the entire country (SABAP2 unpublished data). This SABAP2 coverage was better than the average for every province except Gauteng, and represents the best coverage of a large area away from a major city.

In SABAP1 Secretarybirds were observed in 1 155 QDGCs, but in SABAP2, as at 19 April 2013, the species had only been observed in 623 (53.9%) of these QDGCs. It had, however, been observed in 107 new QDGCs in SABAP2. The provinces with the largest proportion of QDGCs in which Secretarybirds were recorded for SABAP1 but where they had not yet been seen in SABAP2 were Limpopo (63.3%) and Northern Cape (60.4%), and the province with the largest proportions of new QDGCs for the species in SABAP2 was Western Cape (16.5%).

### CAR project

In total, 2 667 Secretarybirds were recorded with habitat data in summer CAR surveys, with an average group size of 1.37 birds, and 2 793 in winter surveys, with an average group size of 1.39 birds. Secretarybirds had been recorded on sufficient numbers of routes for 16 precincts to be included in the analyses of habitat selection and use. Based on the Jacobs index, Secretarybirds preferred natural habitats in both summer and winter in 13 precincts and in summer only in the remaining three precincts (Table 2). Preference for natural habitats was greater in summer than in winter in 12 of the 16 precincts.

**Table 2:** Habitat selection by Secretarybirds observed in the Coordinated Avifaunal Roadcounts (CAR) project in the 16 precincts for which there were sufficient data, in summer (S) and winter (W) counts.

Precinct	Season	Jacobs index	Natural	Transformed	Sign test *p* value
Eastern Cape Coastal	S	0.759	6	2	0.157
	W	0.663	10	0	0.002**
Eastern Cape Karoo	S	0.387	19	2	<0.001***
	W	0.715	22	1	<0.001***
Eastern Karoo	S	0.136	64	2	<0.001***
	W	–0.058	49	2	<0.001***
Gauteng	S	0.220	9	3	0.083
	W	0.380	16	4	0.007**
Mpumalanga	S	0.481	6	1	0.059
	W	0.028	6	5	0.763
North-eastern Eastern Cape	S	0.277	17	5	0.011*
	W	–0.028	13	10	0.532
North-eastern Free State	S	0.454	21	8	0.016*
	W	0.430	36	12	0.001**
North-western Free State	S	0.923	14	0	<0.001***
	W	0.343	16	8	0.102
Northern KwaZulu-Natal	S	0.654	17	2	0.001**
	W	0.307	14	6	0.074
Overberg	S	0.223	21	11	0.077
	W	0.477	20	12	0.157
Southern Free State	S	0.606	93	12	<0.001***
	W	0.656	106	5	<0.001***
Steenkampsberg	S	0.055	8	1	0.020*
	W	–0.061	2	5	0.257
Southern KwaZulu-Natal	S	0.558	18	3	0.001**
	W	0.489	20	3	<0.001***
Swartland	S	0.175	6	5	0.763
	W	0.160	6	7	0.782
Wakkerstroom	S	0.429	6	3	0.317
	W	0.344	9	1	0.011*
Western KwaZulu-Natal	S	1.000	9	0	0.003**
	W	0.522	6	1	0.059

Jacobs index *D* values indicate selection for natural habitats if positive, and for transformed land if negative. The Natural and Transformed columns give the numbers of routes on which Secretarybirds showed a preference for natural or transformed habitats respectively. Sign test *p* values refer to tests of whether the number of routes with positive or negative Jacobs index *D* values was significantly different from that expected if zero selection had been shown.

The number of routes in each precinct on which Secretarybirds showed a preference for natural habitats was greater than the number on which transformed habitats were preferred in all cases except in Steenkampsberg and Swartland in winter; this difference was statistically significant in 18 of the 32 precinct-season combinations (sign tests; Table 2).

**Figure 4:**
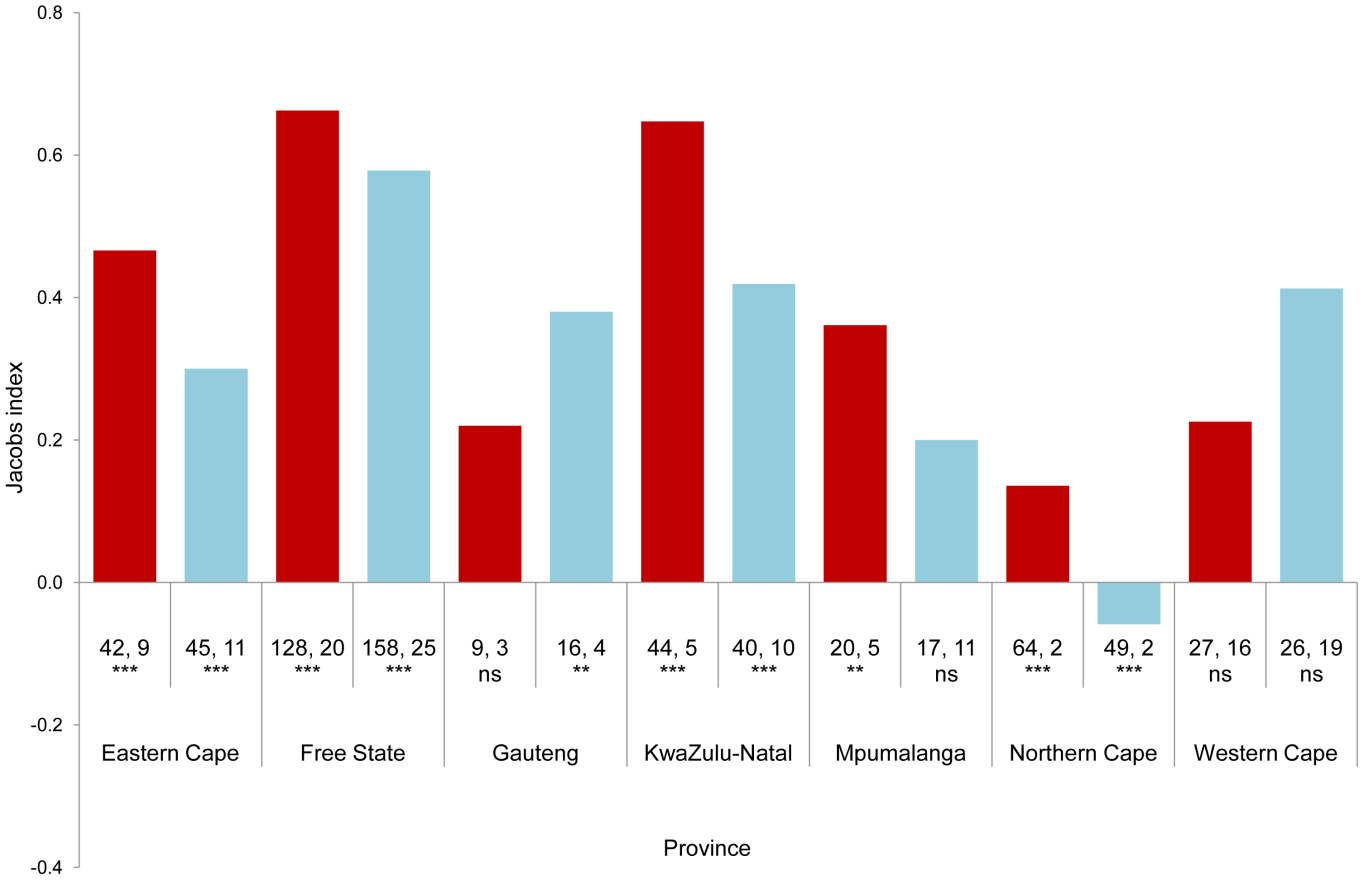
Habitat selection by Secretarybirds observed in the CAR project, with CAR routes grouped by province. Jacobs index values between –1 and zero indicate selection for transformed habitats, and between zero and +1 indicate selection for natural habitats. Each province is separated into summer (red bars) and winter (blue bars). Numbers below the bars are the number of routes on which Secretarybirds showed a preference for natural or transformed habitats, respectively. These numbers were tested using the sign test and the level of statistical significance is indicated beneath the numbers, as follows: *** for *p*<0.001, ** for *p*<0.01 and ns (not significant) for *p*>0.05.

When routes were pooled by province rather than precinct, the species' preference for natural habitats was strongest in the Free State (both seasons) and KwaZulu-Natal (summer), with Jacobs index values of c. 0.6 (Table 3, Figure 4). The remaining province-season combinations showed a preference for natural habitats ranging between 0.1 and 0.5, with the exception of the Northern Cape in winter, when the birds showed a slight preference for transformed habitats (Jacobs index value of 0.06).

**Table 3:** Habitat selection by Secretarybirds observed in the CAR project in the seven provinces in which the CAR project operates, in summer (S) and winter (W) counts.

Province	Season	Jacobs index	Natural	Transformed	Sign test *p* value
Eastern Cape	S	0.466	42	9	<0.001***
	W	0.300	45	11	<0.001***
Free State	S	0.662	128	20	<0.001***
	W	0.578	158	25	<0.001***
Gauteng	S	0.220	9	3	0.083
	W	0.380	16	4	0.007**
KwaZulu-Natal	S	0.647	44	5	<0.001***
	W	0.419	40	10	<0.001***
Mpumalanga	S	0.361	20	5	0.003**
	W	0.200	17	11	0.257
Northern Cape	S	0.136	64	2	<0.001***
	W	–0.058	49	2	<0.001***
Western Cape	S	0.226	27	16	0.093
	W	0.413	26	19	0.297

Data used for this analysis were identical to those used for the analysis presented in Table 2. Columns are as for Table 2.

**Figure 5:**
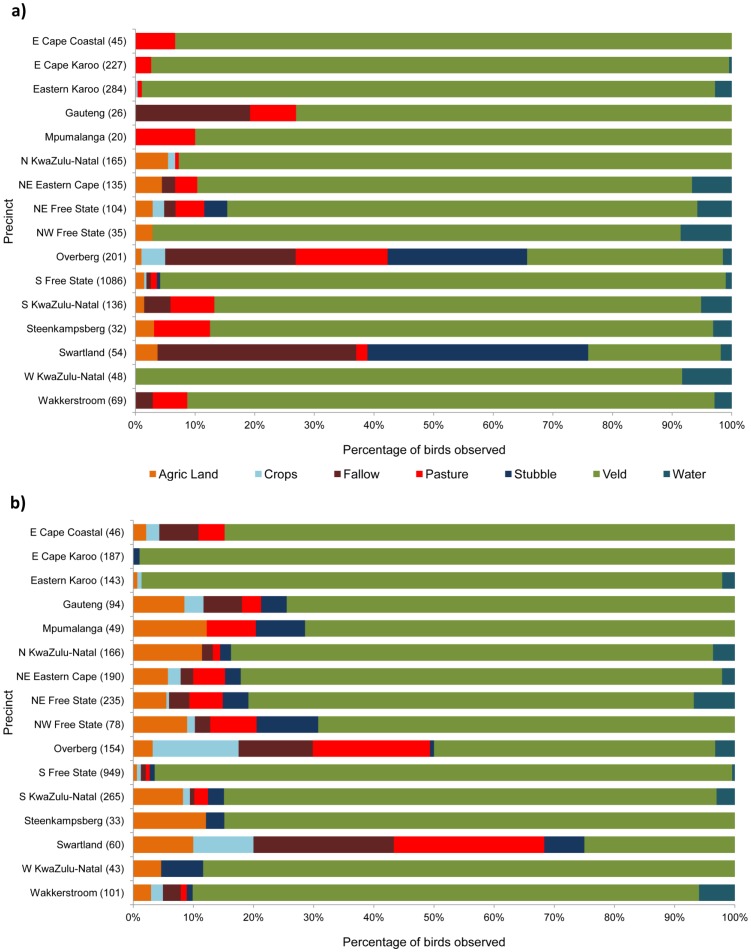
Habitat use by Secretarybirds observed in the CAR project in (a) summer and (b) winter. Data are presented for the 16 precincts for which there were sufficient data for habitat selection analyses. Numbers in brackets following the precinct name give the total number of birds recorded in that precinct in surveys conducted in that season, from the start of counts in that precinct until summer 2013. “Agric Land” consists mainly of ploughed, burned and mowed land but also includes other miscellaneous types of farmland; “stubble” indicates harvested crop fields, “crops” includes all cultivated crops, orchards and vineyards, and “veld” indicates natural vegetation.

Graphical summaries of the habitat use data confirmed that the habitat in which Secretarybirds were most frequently observed was “veld”, i.e. natural vegetation (Figure 5). In summer at least 80% of sightings were in veld in 12 of the 16 precincts, and in winter that number was 10. The striking exceptions were the two Western Cape precincts, Swartland and Overberg, where 76% and 66% respectively of summer sightings were in agricultural habitats, and for winter these figures were 75% and 50%. Habitat types other than veld that were favoured in these precincts in summer were crop stubbles, fallow land, and pasture. In winter the transformed habitat types most used were pasture, fallow land, and crop fields.

## Discussion

The methods developed here represent an important new approach to the analysis of bird atlas data and habitat use data. These methods are applicable to other species covered by SABAP and CAR, and could easily be adapted for use with similar datasets collected in other parts of the world and for other species. They are, however, exploratory and innovative, and necessarily come with caveats regarding their interpretation. That said, our SABAP-related findings have received confirmation through an analysis of reporting rate changes for bird families throughout South Africa [32]. This study modelled bird families in relation to the proportion of QDGCs in their range in which reporting rates had increased between SABAP1 and SABAP2. Sagittariidae (a single-species family that includes only the Secretarybird) was fourth lowest in a list of 51 families. This implies that the Secretarybird is faring particularly badly in comparison with the majority of other South African bird species.

The assumption that changes in atlas reporting rates reflect changes in abundance has not been rigorously tested for this species or in the habitats in which it occurs. The closest we have to a test of this assumption is presented by Griffioen [20], who found that the abundance of Australian birds could be estimated from atlas reporting rates. This work has informed our own (see below).

Apart from changes in abundance, factors that could lead to changes in reporting rates include changes in: bird behaviour; observer skill and effort; vegetation; and project design. Bird behaviour is unlikely to be a factor when comparing data covering two periods of several years' duration. While individual observer skill and effort naturally vary among atlas lists, the data set for each atlas project is so large that it is highly unlikely that there is a consistent difference either way between the two projects. Changes in vegetation may be a factor, especially in areas where bush encroachment has been substantial between the two atlas projects (discussed below). However, this species is known to avoid densely vegetated habitats [2], so any declines in reporting rates in areas where vegetation density has increased are likely to reflect actual declines in abundance. Project design did change between the two atlas projects, but if this were to lead to a bias in reporting rates, we would expect to see similar results for all similar species, but this is not the case [3], [32].

Our use of standard approaches to testing for statistical significance in the difference between two proportions in this analysis is a first attempt at developing some measure of how real a change in reporting rates is likely to be. It should therefore be interpreted with caution, and is subject to refinement. It is also important to remember that any abundance-related inferences we draw from changes in reporting rates are limited to the direction of the change; at this stage we can say nothing with confidence about the magnitude of such change.

The habitat selection analysis is unfortunately limited by the habitat availability information that is available. The National Land-Cover maps are produced every five to 10 years, primarily from satellite photographs, and are designed to provide information that will be useful and relevant for several years. They therefore do not distinguish between seasonally changing habitat types, such as crop fields and crop stubbles. In contrast, the habitat use data collected in CAR surveys record habitats that are relevant to birds on a local and seasonal scale, so the distinction between crop fields and crop stubbles is highly relevant. The habitat categories in the two datasets therefore had to be grouped such that they could be compared, and the natural/transformed classification was the only grouping that satisfied this requirement. The CAR data distinguish well between transformed (crops, pastures, ploughed fields, etc.) and modified or natural land (“veld”), and the National Land-Cover maps essentially do the same (cultivated land vs. natural land). Although we recognise that as far as Secretarybirds are concerned the distinction between transformed and modified land may in some cases be arbitrary, our results suggest that overall this is not the case.

### SABAP data

The consistent decrease in bird atlas reporting rates for the Secretarybird in a large proportion of QDGCs (Figures 1 and 3) suggests a decrease in abundance across most of its South African range between the two atlas projects (1991 and 2007). Worryingly, there were almost uniformly decreased reporting rates throughout the Kruger National Park, South Africa's largest formal conservation area, which is often considered to be an important refuge for many large, threatened bird species, especially raptors [33]. This area was also well covered in terms of the number of atlas lists submitted in both atlas projects, which adds considerable weight to the conclusion that this decrease in reporting rates represents a real decrease in abundance. The large size of QDGCs relative to most other formally protected areas precluded a similar analysis for other protected areas. We envisage, however, that analysis of trends in the finer-scale SABAP2 data will become possible for these smaller areas when data have accumulated for several more years.

In the Kruger National Park it is possible that changes in the predominant vegetation characteristics have brought about this decrease. The abundance of small to medium-sized shrubs and bushes increased over much of the park between 1940 and 1998 [34] and this trend is likely to have continued since 1998, based on findings for other parts of South Africa and other conservation areas [35]. This may have rendered much of the habitat unsuitable for Secretarybirds, which prefer open habitats. It has been suggested that the increasing atmospheric carbon dioxide concentration has already caused and will continue to cause a general increase in woody vegetation at the expense of grassland and savannas across South Africa and globally [36]–[38], so this may constitute a major threat to open habitat species such as the Secretarybird. Support for this hypothesis is provided by a study conducted in Swaziland, in which bush encroachment (increase in dense woody vegetation in previously open habitat) was confirmed to be occurring and was found to have a strong effect on bird abundances [39]. All the species that declined significantly in abundance were associated with open habitats, and those that increased were associated with closed habitats. This study took place over just 10 years (1998–2008), implying that it is not infeasible for such a phenomenon to have occurred between SABAP1 and SABAP2 with Secretarybirds.

There were four other areas where a large proportion of QDGCs showed reduced reporting rates, concentrated in the mesic Grassland and Savanna biomes in the east of the country: (i) around the junction of the Limpopo, North-West, Mpumalanga and Gauteng provincial borders (Savanna); (ii) north-east Free State and north-west KwaZulu-Natal (Grassland); (iii) north-east KwaZulu-Natal (Savanna), and (iv) south-west KwaZulu-Natal, the foothills of the Drakensberg mountains (Grassland) (Figure 1). Based on examination of the land-cover and vegetation maps used in this study [23]–[26], reasons for declines in these areas may include habitat transformation for cultivation (areas i, ii and iii), extensive land degradation (areas i, ii and iii), urbanisation and human population pressure (areas i and ii) and afforestation (areas ii and iv). However, as in the Kruger National Park, widespread bush encroachment may be the most important threat to Secretarybirds. In addition to increasing atmospheric carbon dioxide concentrations, possible reasons for this bush encroachment include fire suppression (mainly by commercial farmers to limit liability for damage caused to neighbouring farms) and abandonment of small maize fields in communal farming areas [35], [36], [38], [40]–[43].

The relationship between reporting rates and abundance is monotonic; the mathematical form that appears to fit best is the one described by Griffioen [20]. This relationship varies between species, so inter-species comparisons cannot be made. Griffioen's [20] model suggested a close-to-linear relationship between abundance and reporting rates less than 60% [3], [20]. Virtually all reporting rates for Secretarybirds were less than 60% and we believe that our results, based on reporting rates, are closely related to abundance. Apparent declines in KwaZulu-Natal, at least, have some tentative support from data held by the provincial conservation body, Ezemvelo KZN Wildlife. Annual aerial surveys are conducted in that province, along standardised routes, primarily for monitoring cranes, but other species are also recorded. The numbers of Secretarybirds seen in these aerial surveys have decreased by more than 50% between 2009 and 2012 (EKZNW unpublished data), although the data series is too short and too sparse for statistical analysis.

The large proportion of QDGCs in Northern Cape in which Secretarybirds were recorded in SABAP1 but not in SABAP2 (Table 1) may reflect the generally poor coverage of this province (7.8 lists per QDGC), in addition to a possible reduction in range. Secretarybirds have large home ranges and in many arid areas tend to be nomadic when not breeding [2], so the probability of seeing them in a pentad on any one visit is small. Coverage of Limpopo and North West, which also had particularly large proportions of such QDGCs, was however much better, at 34.6 and 34.5 lists per QDGC respectively, so the results for these provinces represent more reliable evidence for reductions in range.

The province with the largest proportion of apparently newly occupied QDGCs (Western Cape) is characterised by a large proportion of transformed land. The natural vegetation in much of this province is fynbos, most of which has been transformed for agricultural use. In common with two other large terrestrial bird species with similar habitat preferences (Blue Crane and Denham's Bustard *Neotis denhami*) [3], Secretarybirds appear to have adapted well to agricultural land in the Fynbos biome. This is borne out here by the CAR project's habitat use data (Figure 5). Secretarybirds hunt by walking large distances and kicking or stamping on prey that is exposed on the ground [2]. This mode of hunting is more effective in low vegetation and on bare ground than in the dense, bushy vegetation typical of fynbos.

### CAR habitat use data

The habitat selection analysis indicates that on the whole, Secretarybirds preferred natural habitats to transformed land (Tables 2 and 3, Figure 4). The main exceptions were Eastern Karoo (winter), North-eastern Eastern Cape (winter) and Steenkampsberg (both seasons). The Eastern Karoo result may seem surprising, because the sign tests indicate that a preference for natural habitats was expressed on a highly significant majority of routes. However, the proportion of available habitats that were transformed was extremely low (less than 2%; unpublished data), so a similarly low proportion of birds needed to be seen on transformed land for the Jacobs index to indicate that they were expressing a preference for that habitat. The karoo biomes are semi-desert, and in general the land is too dry for cultivation or pastures. Where crops or pastures are grown, the land is usually irrigated, which may cause the biomass of potential Secretarybird prey species (rodents, reptiles, small birds, insects) to increase. If this is the case, it would make sense for Secretarybirds to prefer transformed habitats where they are available in the karoo. In fact Secretarybirds are thinly distributed in the karoo, preferring grassland and savanna habitats [2], so the small amount of cultivation in the karoo may actually have made the region somewhat more hospitable for the species.

In the North-eastern Eastern Cape and Steenkampsberg precincts the transformed habitats in which Secretarybirds were seen almost all consisted of bare land or low vegetation (Figure 5), where prey visibility and the ability to walk unimpeded were probably improved relative to the natural habitat (grassland). That said, birds still showed a slight preference for natural habitats in summer, and exhibited very close to neutral habitat selection in winter.

In the two Western Cape precincts, Overberg and Swartland, at least 50% of birds were seen in transformed habitats in both seasons (Figure 5). Because of the large percentage of transformed habitats in these precincts, this did not translate into a preference for those habitats. It does, however, demonstrate that the species has been able to adapt to transformed habitats in this biome.

The increased use by Secretarybirds of transformed habitats in winter in most precincts in the summer rainfall region was unexpected. It is unlikely to be related to breeding, because chicks are altricial and remain in the nest (which is usually placed on top of a small tree) until they fledge [2]. The capacity of vegetation to shelter and hide young birds, therefore, does not apply to this species. Secretarybirds have been observed using alien tree species and even electricity pylons to nest, and in many vegetation types apparently suitable nesting trees are often more abundant in transformed areas than in the natural vegetation. Nest tree availability therefore does not provide a plausible explanation for the observed seasonal changes in habitat preferences. Changes in natural vegetation structure are also unlikely to explain the change in habitat use, because in most of the country this vegetation would generally be denser and more difficult to walk in, and prey visibility would be poorer, in summer than in winter. In the winter rainfall region of the Fynbos biome there is unlikely to be any noticeable difference in natural vegetation between seasons. However, the change from cultivated crops to crop stubbles in summer rainfall areas may make some transformed habitats more attractive to Secretarybirds in winter. Tentative confirmation for this is provided by the reverse trend in winter rainfall areas (Tables 2 and 3, Figure 4), where the timing of crop harvesting is also reversed. Changes in prey abundance may also partially explain the seasonal change in habitat use. In most of South Africa winters are dry [44], so it is possible that in transformed habitats prey abundance is relatively higher in winter because of irrigation and/or the application of fertiliser, which keeps vegetation biomass artificially high. This finding requires further exploration.

## Conclusions

Bird atlas data for South Africa suggest that the Secretarybird population declined across most of the country, and particularly severely in the Kruger National Park, between the early 1990s and the late 2000s–early 2010s. Although these findings are of concern, this time period is relatively short, and in some less accessible areas coverage for SABAP2 had not yet reached desirable levels. A longer data series and broader coverage are required before we would be able to state with confidence that the species population had declined significantly in South Africa.

Habitat data from the CAR project show that this species tends to avoid transformed habitats across most of the area covered by this project. One cause of the probable decrease, then, is likely to be habitat loss due to anthropogenic land transformation. This, however, is unlikely to be the case in areas such as the Kruger National Park, and in this and other areas widespread bush encroachment is probably an important threat to Secretarybirds. Other potential causes for decreases in this species include powerline and fence collisions, occasional inadvertent poisoning by insecticides, and human disturbance. The Endangered Wildlife Trust has records of 62 powerline collisions and two electrocutions for the period 1996–2012, but only two records of suspected poisoning incidents (EWT unpublished data). There is no obvious trend in these data, but the former is not an insignificant number, especially considering that the percentage of powerline collision incidents that are reported is low [45]. Despite the general lack of negative beliefs about Secretarybirds among commercial farmers and in traditional African belief systems, human disturbance is suspected to be one of the main causes of the species' decline throughout the rest of its range [1], [2], [46]. Because the species is wide-ranging [2], this effect in neighbouring countries may affect the South African population as well. Human disturbance was the cause of the failure of at least four breeding attempts out of 15 studied in what is now Gauteng in 1977–1988 [47], so this may also be a significant threat within South Africa. It is recommended, therefore, that a publicity campaign about protecting this species (particularly its nests) is launched. In addition, an in-depth study of the movements, habitat use and general ecology of the species is recommended, to increase our knowledge of the species and the best ways in which to conserve it.

These findings would have been impossible were it not for the substantial volumes of citizen science data we analysed. We have developed new methods for analysing citizen science data, in particular bird atlas data from two time periods. These are likely to be applicable to many similar projects elsewhere in the world. Data from these projects are of great value in detecting population trends and understanding the ecology of many poorly studied and difficult-to-census species. We strongly recommend that any new long-term monitoring projects are designed with this type of data analysis in mind. This will greatly facilitate the extraction of useful and reliable information from these citizen science project datasets.

## Supporting Information

Figure S1 SABAP comparison map for the Secretarybird, extracted 19 April 2013. Colour coding of QDGCs as per Figure 1. Additional data presented here are the reporting rates and *Z* values used to colour-code QDGCs. The upper number in each square is the SABAP1 reporting rate, the middle number is the SABAP2 reporting rate, and the lower number is *Z*.
(TIF)
