# Supplementary figures and images for: Secretarybird Sagittarius serpentarius Population Trends and Ecology: Insights from South African Citizen Science Data
Source: PLoS One. 2014 May 9;9(5):e96772. doi: 10.1371/journal.pone.0096772 (PMC4016007; doi:10.1371/journal.pone.0096772)

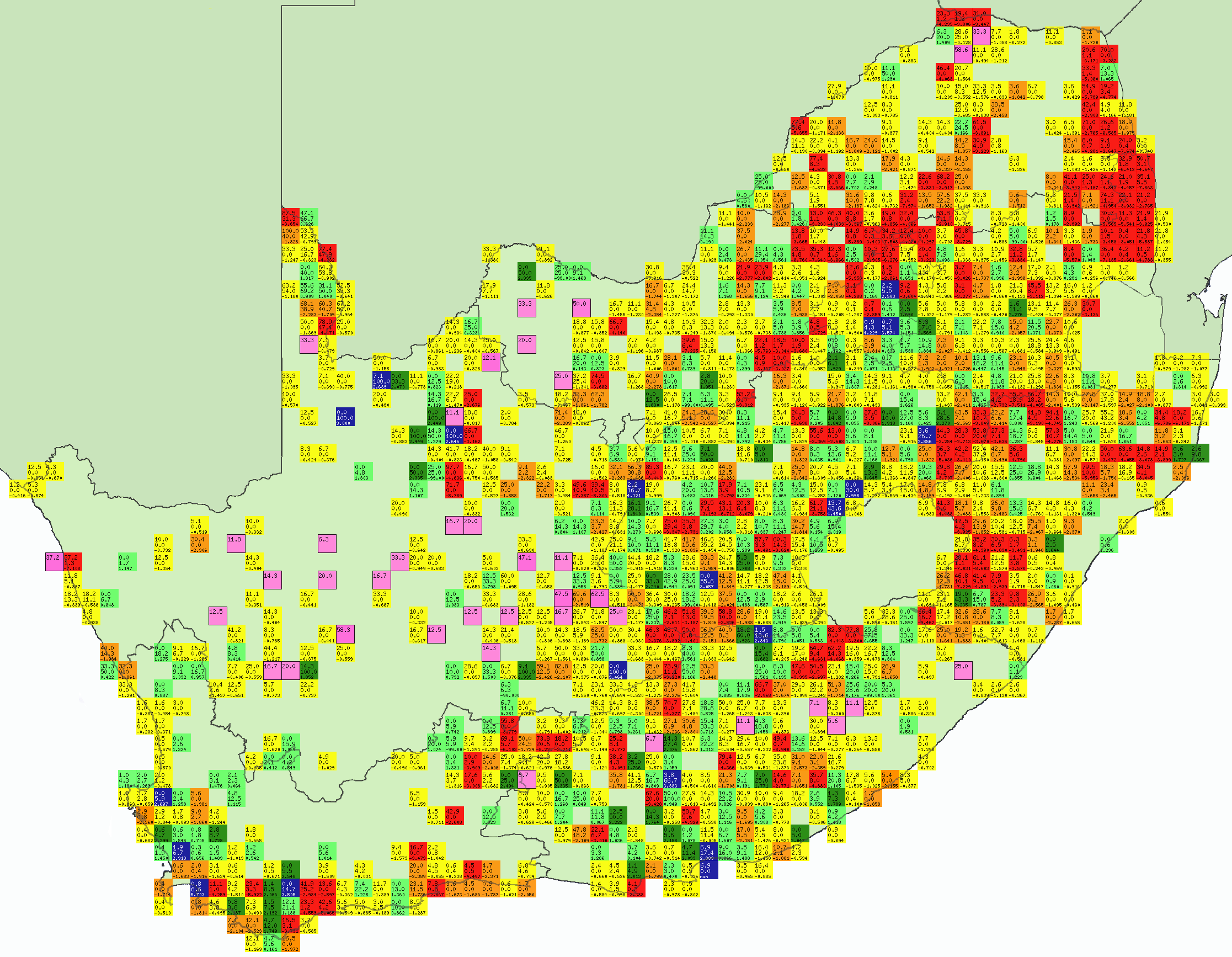

Supplement: Figure S1 — SABAP comparison map for the Secretarybird, extracted 19 April 2013. Colour coding of QDGCs as per Figure 1. Additional data presented here are the reporting rates and Z values used to colour-code QDGCs. The upper number in each square is the SABAP1 reporting rate, the middle number is the SABAP2 reporting rate, and the lower number is Z. (TIF) [file pone.0096772.s001.tif]
